# Beat-to-Beat Nystagmus Differentiation Between Benign Paroxysmal Positional Vertigo (BPPV) and Vestibular Migraine (VM): A Cross-Sectional Videonystagmographic Study From Northern India

**DOI:** 10.7759/cureus.116148

**Published:** 2026-09-12

**Authors:** Pratishtha Sachan, Srinivas Dorasala, Utkarsh Bansal, Abhishek Gupta, Mili Sengar, Vivek Verma, Beena Sachan

**Affiliations:** 1 Otolaryngology, Pravigya Ear, Nose, Throat (ENT) and Vertigo Clinic, Lucknow, IND; 2 Otolaryngology, Yenepoya University, Mangalore, IND; 3 Pediatrics, Hind Institute of Medical Sciences, Barabanki, IND; 4 Community Medicine, Prasad Institute of Medical Sciences, Lucknow, IND; 5 Community Medicine, Dr. Ram Manohar Lohia Institute of Medical Sciences, Lucknow, IND

**Keywords:** crescendo-decrescendo pattern, diagnostic differentiation, dizziness, migraine prophylaxis, positional nystagmus, positional vertigo, repositioning maneuvers, slow phase velocity, vestibular migraine, videonystagmography

## Abstract

Background

Benign paroxysmal positional vertigo (BPPV) and vestibular migraine (VM) are among the most common causes of vertigo encountered in clinical practice. Both conditions can present with similar symptoms, such as dizziness and positional vertigo, yet their underlying mechanisms and management differ significantly. Misdiagnosis can lead to ineffective or inappropriate treatment.

Objective

This study aims to evaluate the characteristics of beat-to-beat positional nystagmus in patients with BPPV and VM using videonystagmography (VNG) and to identify distinguishing features of nystagmus that facilitate accurate diagnosis and treatment.

Methods

A cross-sectional observational study was conducted at a tertiary care center in Lucknow, India. Of 343 patients screened for positional vertigo, 110 patients who met the inclusion criteria were enrolled. Patients were divided into two groups based on clinical and diagnostic criteria: BPPV (n=57) and VM (n=53). All patients underwent a comprehensive vestibular assessment using VNG, with specific attention to slow-phase velocity (SPV), amplitude, direction, and duration of nystagmus during various positional tests. Statistical analysis was conducted using descriptive statistics and t-tests.

Results

The study revealed distinct differences in nystagmus characteristics between patients with BPPV and VM. Patients with BPPV demonstrated a characteristic crescendo-decrescendo pattern in SPV, indicating a mechanical etiology linked to canalith kinetics. In contrast, patients with VM showed a higher initial SPV with a more sustained, constant pattern of nystagmus, suggestive of central vestibular hyperexcitability. Peak nystagmus amplitude and SPV were significantly higher in BPPV (mean peak amplitude: 11.55° vs. 5.64°, p=0.02; mean peak SPV: 21.16°/s vs. 13.10°/s, p=0.005). Positional testing demonstrated distinct patterns between the two groups. The Dix-Hallpike (DH) and supine head-hanging (head extension) positions were more frequently positive in patients with BPPV, whereas the pitch-forward (head flexion) position more frequently elicited nystagmus in patients with VM.

Conclusion

VNG-based assessment of beat-to-beat nystagmus characteristics can be a supportive, objective method for differentiating BPPV from VM in difficult cases. Accurate differentiation is crucial for guiding appropriate therapeutic strategies, including repositioning maneuvers for BPPV and migraine prophylaxis for VM.

## Introduction

Benign paroxysmal positional vertigo (BPPV) is recognized as the most common peripheral vestibular disorder [1,2], primarily caused by dislodged otoconia migrating into the semicircular canals. These otoconial particles stimulate the cupula during specific head movements, resulting in transient vertigo and positional nystagmus. The condition is typically diagnosed using positioning maneuvers such as the Dix-Hallpike (DH) maneuver and is treated effectively with canalith repositioning maneuvers such as the Epley maneuver [3,4].

Vestibular migraine (VM), on the other hand, is a central vestibular disorder [5,6] associated with migraine pathophysiology. Patients often present with dizziness or vertigo in the absence of headache or with subtle migrainous symptoms, making clinical diagnosis challenging. Although VM may mimic BPPV by eliciting positional nystagmus, the exact underlying pathophysiological mechanism remains unknown [7].

Differentiating between BPPV and VM is essential because of their different treatment modalities. While BPPV typically resolves with repositioning maneuvers, VM requires migraine prophylaxis, dietary modifications, and central vestibular suppression [8]. In this context, videonystagmography (VNG) provides an objective tool for analyzing eye movements and nystagmus characteristics that can aid in accurate diagnosis [9].

This study aims to explore the beat-to-beat characteristics of nystagmus, including amplitude, slow-phase velocity (SPV), direction, and duration, among patients with BPPV and VM using VNG as the diagnostic modality. The primary objective of the present study was to differentiate these disorders based on characteristic nystagmographic patterns rather than on migraine-associated clinical symptoms alone.

## Materials and methods

Study design and setting 

A cross-sectional, observational study was conducted at a tertiary care hospital in Lucknow, India.

Study population****

Patients presenting to the ENT outpatient department with complaints of vertigo or dizziness were included in the study. Data collection was conducted on alternate clinic days (Monday, Wednesday, and Friday) for a period of one year, from June 2024 to May 2025. On each study day, eligible participants were selected using systematic random sampling by enrolling every second eligible participant after a random start.

To be included in the study, participants had to be at least 18 years of age, present with a primary complaint of positional vertigo, and exhibit positional nystagmus elicited by VNG testing. Additionally, they had to have been residents of Lucknow for at least six months.

All patients underwent the appropriate canalith repositioning maneuver. Patients who showed complete resolution of symptoms and positional nystagmus following the repositioning maneuver were classified as confirmed BPPV. Patients who did not improve after appropriate repositioning maneuvers but subsequently responded to VM prophylactic therapy were classified as having VM presenting with positional vertigo [10].

Participants were excluded if they had received betahistine, cinnarizine, or any vestibular suppressant before undergoing VNG, as these medications may modify vestibular responses and alter positional nystagmus patterns [11].

Patients with atypical BPPV were excluded [12]. Individuals fulfilling the Bárány Society/International Headache Society diagnostic criteria for VM, including a history of migraine with characteristic symptoms such as headache, photophobia, phonophobia, or visual aura, were also excluded [13]. Patients with psychiatric illnesses or other central vestibular disorders were excluded from the study.

Participants were excluded from the study if they had central nervous system disorders other than VM or a history of recent head trauma. Individuals using ototoxic medications and patients who were uncooperative or critically ill were also considered ineligible for participation.

Over a period of one year, 343 patients were approached; 110 met the inclusion and exclusion criteria and were enrolled: 57 were diagnosed with BPPV and 53 with VM.

Diagnostic workup 

VNG was performed using the same device (Equi COG) in all patients. Balance Eye is a binocular VNG system that is Class IIa European CE certified. It provides eye-movement recording with vision allowed and denied. Before each recording, the VNG system was calibrated according to the manufacturer's protocol. Participants were instructed to fixate on visual targets presented at predefined horizontal and vertical positions while keeping their heads stationary, allowing accurate calibration of eye position relative to gaze angle. Calibration was repeated whenever tracking accuracy was suboptimal or after repositioning of the goggles to ensure reliable eye-movement recordings throughout the examination [14]. We evaluated oculomotor activities such as saccades, smooth pursuit, gaze with and without fixation, and responses to horizontal high-frequency headshake, hyperventilation, lateral canal head impulse testing, and positional tests. In the primary gaze position, spontaneous nystagmus was recorded using VNG. SPV (°/s) and nystagmus amplitude (°) were measured and analyzed.

All VNG examinations were performed by a single trained examiner. Owing to the study design, the examiner was not blinded to the participants' clinical diagnoses.

All participants underwent positional testing using VNG: DH (right and left) [15], McClure-Pagnini [16], pitch forward/backward, yaw test (right and left), and roll test (right and left) [17].

Nystagmus was analyzed for direction (Upbeat (UB), Downbeat (DB), Right-beating (RB), or Left-beating (LB)); onset latency and fatigability; amplitude (angular excursion of the eye during the slow phase, measured in degrees) at the beginning, peak, and last beat; and SPV (measured in degrees per second) across different positional tests.

Data analysis 

Statistical analysis was performed using SPSS software, version 24.0 (published in 2016 by IBM Corp., Armonk, NY, USA). Descriptive statistics and t-tests were used. A p-value <0.05 was considered statistically significant.

## Results

A total of 343 patients were screened for positional vertigo, and 110 patients who met the inclusion and exclusion criteria were enrolled. Patients were divided into two groups based on clinical and diagnostic criteria: VM (n=53, 48.2%) and BPPV (n=57, 51.8%).

Table 1 presents the outcomes of various positional tests performed on patients in the two groups. In the Pitch Forward test, 45.3% (24/53) of VM patients showed a positive response, whereas 33.3% (19/57) of BPPV patients were positive. In the DH Supine Head Ext Rt test, 45.3% (24/53) of VM patients tested positive, while 45.6% (26/57) of BPPV patients were positive, suggesting a similar response rate in both conditions. Some tests, such as MP Supine Head Neutral 2, showed very low positivity rates across both groups, with 5.7% (3/53) in VM and 0% (0/57) in BPPV, suggesting limited diagnostic utility for this maneuver. The table aids in identifying which positional maneuvers are more likely to provoke a response in patients with VM versus BPPV, helping to differentiate between the two vestibular disorders based on positional test findings.

**Table 1:** Comparison of positional test outcomes between patients with vestibular migraine (VM) and benign paroxysmal positional vertigo (BPPV). VM: Vestibular migraine; BPPV: Benign paroxysmal positional vertigo; DH: Dix-Hallpike; MP: McClure-Pagnini; Lt: Left; Rt: Right; Ext: Extension; Lat: Lateral. Data are presented as n (%). Pearson's χ² test was used for comparisons with adequate expected cell counts, and *Fisher's exact test was used for comparisons with small expected cell counts, including those containing a zero cell. A p-value <0.05 was considered statistically significant.

Test	VM (n=53), n (%)	BPPV (n=57), n (%)	χ² value	p-value
Pitch Forward	24 (45.3)	19 (33.3)	1.647	0.199
Pitch Backward	21 (39.6)	19 (33.3)	0.469	0.493
DH Sit Head Rt 1	8 (15.1)	6 (10.5)	0.516	0.473
DH Supine Head Ext Rt	24 (45.3)	26 (45.6)	0.001	0.972
DH Sit Head Rt 2	5 (9.4)	10 (17.5)	1.534	0.216
DH Sit Head Lt 1	4 (7.5)	7 (12.3)	0.684	0.408
DH Supine Head Ext Lt	15 (28.3)	22 (38.6)	1.304	0.254
DH Sit Head Lt 2	5 (9.4)	7 (12.3)	0.229	0.632
MP Sit to Supine	4 (7.5)	8 (14.0)	1.189	0.275
MP Rt Lat	15 (28.3)	12 (21.1)	0.779	0.377
MP Supine Head Neutral 1	5 (9.4)	9 (15.8)	0.999	0.318
MP Lt Lat	15 (28.3)	16 (28.1)	0.001	0.978
MP Supine Head Neutral 2	3 (5.7)	0 (0.0)	-	0.109*

Table 2 presents a detailed distribution of the type and direction of nystagmus observed during various positional tests in patients diagnosed with VM and BPPV. The table categorizes the direction of nystagmus as UB, DB, RB, or LB. The Pitch Backward test showed a high frequency of UB nystagmus in VM (16/53, 30.2%) compared with BPPV (7/57, 12.3%), indicating sensitivity in detecting central or posterior canal involvement, which may help differentiate between the two conditions. The Pitch Forward test showed a high DB response in VM (14/53, 26.4%) compared with BPPV (7/57, 12.3%), which may help differentiate between the two conditions. The McClure-Pagnini test displayed strong RB and LB nystagmus in both groups, with BPPV showing slightly higher LB counts (8/57, 14%). Some maneuvers, such as the Yaw and Roll tests, showed mixed responses with relatively fewer instances of directional nystagmus, indicating variable diagnostic utility.

**Table 2:** Comparison of nystagmus types between patients with VM and BPPV during positional tests. VM: Vestibular migraine; BPPV: Benign paroxysmal positional vertigo; DH: Dix-Hallpike; MP: McClure-Pagnini; Lt: Left; Rt: Right; Ext: Extension; Lat: Lateral; UB: Upbeat; DB: Downbeat; RB: Right-beating; LB: Left-beating. Data are presented as n (%).

Type of test	VM UB, n (%)	VM DB, n (%)	VM RB, n (%)	VM LB, n (%)	BPPV UB, n (%)	BPPV DB, n (%)	BPPV RB, n (%)	BPPV LB, n (%)
Yaw Lt test	3 (5.7)	1 (1.9)	2 (3.8)	0 (0)	2 (3.5)	1 (1.8)	2 (3.5)	0 (0)
Yaw Rt test	1 (1.9)	0 (0)	1 (1.9)	2 (3.8)	0 (0)	1 (1.8)	0 (0)	0 (0)
Pitch backward	16 (30.2)	3 (5.7)	2 (3.8)	0 (0)	7 (12.3)	0 (0)	7 (12.3)	5 (8.8)
Pitch forward	4 (7.5)	14 (26.4)	2 (3.8)	3 (5.7)	4 (7.0)	7 (12.3)	5 (8.8)	3 (5.3)
Roll Rt	3 (5.7)	5 (9.4)	1 (1.9)	1 (1.9)	4 (7.0)	3 (5.3)	4 (7.0)	1 (1.8)
Roll Lt	3 (5.7)	0 (0)	2 (3.8)	1 (1.9)	4 (7.0)	1 (1.8)	2 (3.5)	2 (3.5)
DH Sit Head Rt 1	2 (3.8)	2 (3.8)	2 (3.8)	2 (3.8)	2 (3.5)	1 (1.8)	3 (5.3)	0 (0)
DH Supine Head Ext Rt	8 (15.1)	7 (13.2)	3 (5.7)	6 (11.3)	10 (17.5)	6 (10.5)	6 (10.5)	3 (5.3)
DH Sit Head Rt 2	2 (3.8)	1 (1.9)	1 (1.9)	1 (1.9)	3 (5.3)	4 (7.0)	2 (3.5)	1 (1.8)
DH Sit Head Lt 1	2 (3.8)	0 (0)	0 (0)	2 (3.8)	3 (5.3)	2 (3.5)	0 (0)	2 (3.5)
DH Supine Head Ext Lt	3 (5.7)	4 (7.5)	0 (0)	8 (15.1)	8 (14.0)	4 (7.0)	3 (5.3)	7 (12.3)
DH Sit Head Lt 2	1 (1.9)	2 (3.8)	0 (0)	2 (3.8)	3 (5.3)	1 (1.8)	2 (3.5)	1 (1.8)
MP Sit to Supine	3 (5.7)	1 (1.9)	0 (0)	0 (0)	2 (3.5)	1 (1.8)	2 (3.5)	3 (5.3)
MP Rt Lat	5 (9.4)	2 (3.8)	2 (3.8)	6 (11.3)	1 (1.8)	1 (1.8)	4 (7.0)	6 (10.5)
MP Supine Head Neutral 1	4 (7.5)	0 (0)	1 (1.9)	0 (0)	4 (7.0)	0 (0)	2 (3.5)	3 (5.3)
MP Lt Lat	4 (7.5)	1 (1.9)	6 (11.3)	4 (7.5)	1 (1.8)	1 (1.8)	6 (10.5)	8 (14.0)
MP Supine Head Neutral 2	1 (1.9)	0 (0)	1 (1.9)	1 (1.9)	0 (0)	0 (0)	0 (0)	0 (0)

Table 3 presents a comparison of vertical nystagmus responses (combining upbeat and downbeat) between the VM and BPPV groups. A significantly higher frequency of positive responses was observed during the pitch-backward maneuver in patients with VM compared with those with BPPV (19/53 (35.8%) vs. 7/57 (12.3%), respectively; χ² = 8.452, p = 0.004). The pitch-forward maneuver was also more frequently positive in the VM group than in the BPPV group (34.0% vs. 19.3%), although this difference did not reach statistical significance (χ² = 3.042, p = 0.081). Similarly, positive responses during the modified positional right lateral maneuver were more frequent in VM than in BPPV (13.2% vs. 3.5%), but the difference was not statistically significant (p = 0.086). No statistically significant between-group differences were observed for the yaw, roll, DH, or modified positional maneuvers.

**Table 3:** Comparison of vertical nystagmus during positional testing between patients with VM and BPPV. VM: Vestibular migraine; BPPV: Benign paroxysmal positional vertigo; DH: Dix-Hallpike; MP: McClure-Pagnini; Lt: Left; Rt: Right; Ext: Extension; Lat: Lateral. Data are presented as n (%). Pearson's χ² test was used for comparisons with adequate expected cell counts, and *Fisher's exact test was used for comparisons with sparse expected cell frequencies. A p-value <0.05 was considered statistically significant.

Test	VM (n=53), n (%)	BPPV (n=57), n (%)	χ² value	p-value
Yaw Lt test	4 (7.5)	3 (5.3)	-	0.709*
Yaw Rt test	1 (1.9)	1 (1.8)	-	1.000*
Pitch Backward	19 (35.8)	7 (12.3)	8.452	0.004
Pitch Forward	18 (34.0)	11 (19.3)	3.042	0.081
Roll Rt	8 (15.1)	7 (12.3)	0.185	0.667
Roll Lt	3 (5.7)	5 (8.8)	-	0.718*
DH Sit Head Rt 1	4 (7.5)	3 (5.3)	-	0.709*
DH Supine Head Ext Rt	15 (28.3)	16 (28.1)	0.001	0.978
DH Sit Head Rt 2	3 (5.7)	7 (12.3)	-	0.324*
DH Sit Head Lt 1	2 (3.8)	5 (8.8)	-	0.440*
DH Supine Head Ext Lt	7 (13.2)	12 (21.1)	1.183	0.277
DH Sit Head Lt 2	3 (5.7)	4 (7.0)	-	1.000*
MP Sit to Supine	4 (7.5)	3 (5.3)	-	0.709*
MP Rt Lat	7 (13.2)	2 (3.5)	-	0.086*
MP Supine Head Neutral 1	4 (7.5)	4 (7.0)	-	1.000*
MP Lt Lat	5 (9.4)	2 (3.5)	-	0.259*
MP Supine Head Neutral 2	1 (1.9)	0 (0.0)	-	0.482*

Table 4 presents a comparison of horizontal nystagmus responses (combining right-beating and left-beating) between the VM and BPPV groups. The pitch-backward maneuver demonstrated a significantly higher frequency of positive responses in the BPPV group compared with the VM group (12/57 (21.1%) vs. 2/53 (3.8%); χ² = 7.382, p = 0.007). The modified positional sit-to-supine maneuver was also positive more frequently in patients with BPPV than in those with VM (5/57 (8.8%) vs. 0/53 (0%)); however, this difference did not reach statistical significance on Fisher’s exact test (p = 0.058). For the remaining positional maneuvers, no statistically significant differences were observed between the VM and BPPV groups.

**Table 4:** Comparison of horizontal nystagmus during positional testing between patients with VM and BPPV. VM: Vestibular migraine; BPPV: Benign paroxysmal positional vertigo; DH: Dix-Hallpike; MP: McClure-Pagnini; Lt: Left; Rt: Right; Ext: Extension; Lat: Lateral. Data are presented as n (%). Pearson's χ² test was used for comparisons with adequate expected cell counts, and *Fisher's exact test was used for comparisons with sparse expected cell frequencies. A p-value <0.05 was considered statistically significant.

Test	VM (n=53), n (%)	BPPV (n=57), n (%)	χ² value	p-value
Yaw Lt test	2 (3.8)	2 (3.5)	-	1.000*
Yaw Rt test	3 (5.7)	0 (0.0)	-	0.109*
Pitch Backward	2 (3.8)	12 (21.1)	7.382	0.007
Pitch Forward	5 (9.4)	8 (14.0)	0.558	0.455
Roll Rt	2 (3.8)	5 (8.8)	-	0.440*
Roll Lt	3 (5.7)	4 (7.0)	-	1.000*
DH Sit Head Rt 1	4 (7.5)	3 (5.3)	-	0.709*
DH Supine Head Ext Rt	9 (17.0)	9 (15.8)	0.028	0.866
DH Sit Head Rt 2	2 (3.8)	3 (5.3)	-	1.000*
DH Sit Head Lt 1	2 (3.8)	2 (3.5)	-	1.000*
DH Supine Head Ext Lt	8 (15.1)	10 (17.5)	0.12	0.729
DH Sit Head Lt 2	2 (3.8)	3 (5.3)	-	1.000*
MP Sit to Supine	0 (0.0)	5 (8.8)	-	0.058*
MP Rt Lat	8 (15.1)	10 (17.5)	0.12	0.729
MP Supine Head Neutral 1	1 (1.9)	5 (8.8)	2.525	0.112
MP Lt Lat	10 (18.9)	14 (24.6)	0.522	0.47
MP Supine Head Neutral 2	2 (3.8)	0 (0.0)	-	0.230*

Table 5 presents a comparative statistical analysis of eye movement amplitudes, specifically Beginning Amplitude (BEG AMP), Peak Amplitude (PEAK AMP), and Last Amplitude (LAST AMP), between the two patient groups. BEG AMP: BPPV patients (mean = 5.06°) had slightly higher initial amplitudes than VM patients (mean = 4.09°), but the difference was not statistically significant (p = 0.11); PEAK AMP: A significant difference was observed (p = 0.02), with BPPV patients showing much higher peak amplitudes (mean = 11.55°) compared with VM patients (mean = 5.64°), suggesting greater eye movement intensity in BPPV during peak episodes; and LAST AMP: The final amplitude was higher in BPPV (mean = 4.24°) than in VM (mean = 3.25°), but the difference was again not statistically significant (p = 0.12).

**Table 5:** Comparison of eye movement amplitudes (BEG, PEAK, and LAST) between patients with VM and BPPV. VM: Vestibular migraine; BPPV: Benign paroxysmal positional vertigo; BEG AMP: Beginning amplitude; PEAK AMP: Peak amplitude; LAST AMP: Last amplitude; LB: Lower bound; UB: Upper bound. Amplitude refers to the angular excursion of the eye during the slow phase, measured in degrees (°). Data are presented as n (%), mean (SD), and 95% CIs (LB, UB). A p-value <0.05 was considered statistically significant.

Parameter	Group	n (%)	Mean (SD)	95% CI, LB	95% CI, UB	t-test value	p-value
BEG AMP (°)	VM	53 (48.2)	4.09 (2.48)	3.41	4.78	2.4†	0.11
	BPPV	57 (51.8)	5.06 (3.78)	4.06	6.07		
PEAK AMP (°)	VM	53 (48.2)	5.64 (5.21)	4.2	7.07	5.1†	0.02
	BPPV	57 (51.8)	11.55 (18.26)	6.7	16.39		
LAST AMP (°)	VM	53 (48.2)	3.25 (2.77)	2.48	4.01	2.3†	0.12
	BPPV	57 (51.8)	4.24 (3.90)	3.21	5.28		

These data imply that while initial and final amplitudes are comparable, BPPV is associated with significantly greater peak eye movement amplitude, which may aid in the clinical differentiation between the two conditions.

Table 6 presents a comparative analysis of three key nystagmus parameters: Beginning SPV (BEG SPV), Peak SPV, and Last Discernible Beat SPV between patients with VM and BPPV. For BEG SPV, the mean value in the VM group (8.77°/s) was significantly higher than that in the BPPV group (1.94°/s), with a p-value < 0.001, indicating a statistically significant difference. For Peak SPV, BPPV patients showed a higher mean (21.16°/s) than VM patients (13.10°/s), and the difference was also statistically significant (p = 0.005), suggesting stronger peak responses in BPPV. For Last Discernible Beat SPV, the mean values were 7.36°/s for VM and 6.74°/s for BPPV, with a p-value of 0.56, indicating no statistically significant difference.

**Table 6:** Comparison of nystagmus parameters between patients with VM and BPPV. VM: Vestibular migraine; BPPV: Benign paroxysmal positional vertigo; SPV: Slow-phase velocity; LB: Lower bound; UB: Upper bound; °/s: Degrees per second. Data are presented as n (%), mean (SD), and 95% CIs (LB, UB). A p-value <0.05 was considered statistically significant.

Parameter	Group	n (%)	Mean (SD)	95% CI, LB	95% CI, UB	F value	p-value
Beginning SPV (°/s)	VM	53 (48.2)	8.77 (5.59)	7.22	10.31	62.7	<0.001
	BPPV	57 (51.8)	1.94 (3.20)	1.09	2.79		
Peak SPV (°/s)	VM	53 (48.2)	13.10 (12.99)	9.52	16.68	8.3	0.005
	BPPV	57 (51.8)	21.16 (16.04)	16.9	25.42		
Last Discernible Beat SPV (°/s)	VM	53 (48.2)	7.36 (5.24)	5.92	8.81	0.33	0.56
	BPPV	57 (51.8)	6.74 (5.88)	5.18	8.3		

Table 7 presents a comparison of mean pulse rate and blood pressure (both systolic and diastolic) measured in the supine and standing positions at 1 minute. The mean pulse rate in the supine position was 74.23 ± 6.57 beats per minute, which showed a slight increase to 74.65 ± 5.06 beats per minute upon standing; however, this difference was not statistically significant (p = 0.415).

**Table 7:** Comparison of pulse rate and blood pressure in supine and standing positions at 1 minute (N=110). Data are presented as mean (SD). A p-value <0.05 was considered statistically significant.

Parameter	Position	Mean (SD)	t-test value	p-value
Pulse rate (beats/min)	Supine	74.23 (6.57)	-0.81	0.415
	Standing at 1 min	74.65 (5.06)		
Systolic blood pressure (mmHg)	Supine	122.05 (7.70)	1.92	0.057
	Standing at 1 min	121.50 (7.46)		
Diastolic blood pressure (mmHg)	Supine	75.75 (6.88)	-0.77	0.441
	Standing at 1 min	76.03 (7.11)		

Similarly, systolic blood pressure decreased slightly from 122.05 ± 7.70 mmHg in the supine position to 121.50 ± 7.46 mmHg upon standing, approaching statistical significance with a p-value of 0.057.

Diastolic blood pressure showed a marginal increase from 75.75 ± 6.88 mmHg in the supine position to 76.03 ± 7.11 mmHg in the standing position, with no statistically significant change (p = 0.441).

Overall, the parameters remained relatively stable with postural changes, indicating no significant orthostatic variation in this sample.

## Discussion

This study explored the diagnostic utility of beat-to-beat nystagmus characteristics in distinguishing BPPV from VM among 110 patients presenting with positional vertigo in Northern India. The findings highlight critical physiological and oculographic distinctions between the two conditions, which are often confused in clinical settings due to overlapping symptoms.

The first major observation relates to nystagmus amplitude and SPV. Patients with BPPV exhibited significantly higher peak amplitudes and peak SPV values compared with VM (p = 0.02 and p = 0.005, respectively). These findings are consistent with von Brevern M et al. [1], who noted that BPPV typically presents with more vigorous and canal-specific responses owing to the mechanical displacement of otoliths within the semicircular canals. In contrast, nystagmus in VM is thought to arise from central vestibular processing dysfunction, which may explain the lower peak responses and higher beginning SPV found in our VM group (p < 0.001). This supports Neuhauser HK et al. [6] and more recent VM diagnostic frameworks [13,18], which emphasize central vestibular hyperexcitability.

Positional test responses also diverged significantly between the two groups. BPPV patients showed greater reactivity in maneuvers such as DH and supine head extension, which aligns with the canalithiasis mechanism, where positional changes cause endolymphatic flow and nystagmus. VM patients, while reactive to some positional changes, showed a more diffuse and inconsistent pattern, suggesting central sensitization rather than true canal involvement. Pitch Backward positional testing was significantly positive, and Pitch Forward positional testing was the most frequently positive position in VM in vertical nystagmus. In horizontal nystagmus, Pitch Backward positional testing was significantly positive in BPPV. Mahrous MM [19] similarly described VM as eliciting less consistent and less position-specific nystagmus, often lacking the latency and fatigability characteristic of BPPV. In our study, some tests, such as MP Supine Head Neutral 2, showed very low positivity rates across both groups, with 5.7% (3/53) in VM and 0% (0/57) in BPPV, suggesting limited diagnostic utility for this maneuver (Table 1). Some maneuvers, such as the Yaw and Roll tests, showed mixed responses with relatively fewer instances of directional nystagmus, indicating variable diagnostic utility (Table 2).

The difference in SPV profiles further strengthens this differentiation. While BPPV showed low initial SPV followed by a sharp peak (indicative of a strong mechanical trigger), VM had higher beginning SPV and a more blunted peak, likely due to centrally mediated vestibular hyperexcitability [13,18].

Interestingly, orthostatic vitals (pulse and blood pressure) did not significantly differ between the groups, suggesting that autonomic instability is not a primary differentiator in this context. This finding partially contrasts with Furman JM and Marcus DA (2012) [20], who noted that some VM patients may exhibit subtle dysautonomia; however, the differences may not always manifest significantly in short-term postural tests.

Overall, the study underscores that BPPV and VM, while clinically overlapping, display distinctly different nystagmus characteristics and test responses. This reinforces the need for VNG in differentiating central from peripheral vertigo and advocates for a shift away from purely history-based diagnosis, especially in resource-equipped clinical settings.

Certain limitations of the study are noteworthy. As this was a single-center study, the generalizability of the results is limited. The study also excluded atypical variants of BPPV and patients with overlapping BPPV and VM. The sample was entirely restricted to a specific population in Northern India, so genetic and environmental predispositions to migraine might not map perfectly onto a global population. Other limitations include potential selection bias, the absence of blinding, possible heterogeneity within the BPPV group, and limited statistical analyses without assessment of diagnostic accuracy.

## Conclusions

This study provides a comprehensive comparison of beat-to-beat nystagmus characteristics in patients with BPPV and VM, emphasizing the diagnostic utility of VNG in distinguishing between these two commonly confused causes of positional vertigo. The data revealed significant differences in nystagmus amplitude, SPV, and positional test responsiveness.

VM patients demonstrated a significantly higher initial beat SPV at the onset of nystagmus. However, the SPV profile showed a relatively blunted peak, with less pronounced amplitude escalation over time.

In contrast, patients with BPPV exhibited a distinct, sharp-peaking pattern characterized by significantly higher peak SPV values and steeper SPV acceleration during the nystagmus episode. Similarly, peak nystagmus amplitude was significantly greater in BPPV than in VM. The pronounced amplitude peak in BPPV reflects greater eye-movement intensity during the maximal phase of the positional response, whereas VM showed a less sharply peaked, more gradual amplitude profile.

These findings suggest that BPPV is characterized by a transient, high-intensity, canal-specific response with marked peaks in both SPV and amplitude, while VM tends to exhibit higher initial SPV followed by a broader, less sharply peaked nystagmus pattern.

These findings highlight the limitations of relying solely on clinical history and physical examination for diagnosis. Objective measurement tools such as VNG can help identify key oculographic features that differentiate central from peripheral vertigo, ultimately leading to more accurate diagnosis and appropriate therapeutic interventions.
